# Journey of Mesenchymal Stem Cells for Homing: Strategies to Enhance Efficacy and Safety of Stem Cell Therapy

**DOI:** 10.1155/2012/342968

**Published:** 2012-06-13

**Authors:** Sung Keun Kang, Il Seob Shin, Myung Soon Ko, Jung Youn Jo, Jeong Chan Ra

**Affiliations:** Stem Cell Research Center, RNL BIO, Gasan-Dong, Geumcheon-Gu, Seoul 153-768, Republic of Korea

## Abstract

Human mesenchymal stem cells (MSCs) communicate with other cells in the human body and appear to “home” to areas of injury in response to signals of cellular damage, known as homing signals. This review of the state of current research on homing of MSCs suggests that favorable cellular conditions and the *in vivo* environment facilitate and are required for the migration of MSCs to the site of insult or injury *in vivo*. We review the current understanding of MSC migration and discuss strategies for enhancing both the environmental and cellular conditions that give rise to effective homing of MSCs. This may allow MSCs to quickly find and migrate to injured tissues, where they may best exert clinical benefits resulting from improved homing and the presence of increased numbers of MSCs.

## 1. Introduction

The promise of regeneration is what has sparked an international effort to expand the field of stem cell research. In particular, the study of mesenchymal stem cells (MSCs) and their effects on cellular degenerative diseases is rapidly increasing. The role of MSCs in the modulation of the immune response, immune system activity, and the body's response to inflammation and disease has been widely studied for many years [1–5]. Multiple studies have demonstrated that cultured MSCs have the ability to differentiate into bone and cartilage [6, 7] as well as other cell types and tissues both *in vitro* and *in vivo* [2, 6, 8]. Among other researchers, Ankrum and Karp demonstrated that MSCs differentiate into osteocytes, adipocytes, neural cells, and vascular endothelial cells [2].

Recent research, however, has shown that the environment plays a crucial role in limiting or expanding the differentiation capacity of MSCs [9–13]. Lavasani et al. [13] demonstrated that muscle stem cells from young mice conferred significant lifespan and healthspan extension in progeroid mice, which have stem cells defective in proliferation and multilineage differentiation. Furthermore, microenvironmental changes regulate the potential of MSCs to differentiate into specific cell types [14–17], and this effect on differentiation seems to be characterized by a variety of factors not yet well understood, such as the length of telomeres of cells in the microenvironment [10, 18]. While bone marrow MSCs (BMMSCs) were shown to have a decreased lifespan, rate of population doubling time [19], actual bone formation as patient age increased [14], adipose tissue-derived MSCs (AdMSCs) do not appear to undergo the same senescence pattern as BMMSCs [10, 18]. Mirsaidi et al. [18] demonstrated that murine AdMSCs derived from senile osteoporotic SAMP6 mice showed maintenance of telomere length, telomerase activity, and osteogenic differentiation. In addition, Chen et al. [10] demonstrated that human AdMSCs from elderly (mean age: 71.4 years) and young (mean age: 36.4 years) donors showed similar increases in proliferation rate, osteogenic differentiation potential, and senescence marker patterns, while BMMSCs from the same cohorts showed reduced proliferation rate, decreased differentiation potential, and increased senescence. Ultimately, however, the relationship between MSCs and their environment is reciprocal. Just as the microenvironmental effects on MSCs can constrict their response to a bodily insult, MSCs can activate or deactivate immune system within the environment [1, 5]. MSCs are sometimes referred to as “balancers” due to the extensive research linking the presence and activity of *in vivo* MSCs and homeostasis [20].

The notion of balance is somewhat circuitous; MSCs appear to both rely upon and cocreate a network that facilitates constant communication between normal and damaged cells in the body [20]. MSCs are dispatched by what might be metaphorically compared to a fire alarm, through a signaling system that has been extensively studied but remains not fully understood. In particular, the factors that trigger MSC responses and the tools required for MSCs to respond in a positive way to a particular insult to the body remain largely unknown [21–25]. To effectively fight the fires in our bodies, an adequate supply of MSCs with high potential are needed to, metaphorically, act as firefighters. Properly culture-expanded and engineered MSCs with enhanced homing capability can ensure removal of the damaged cells and increase the rate of regeneration when the balance is disrupted in the body. Furthermore, strategies to modulate the physiological barrier of blood vessels and the lung, the inflammatory microenvironment of the body, and the chemotactic signals from the damage site will enhance MSC homing. A variety of strategies have been suggested to enhance the homing of MSCs based on their well-known characteristics.

In this paper, we will review the current understanding of MSC migration and discuss strategies for enhancing their trafficking to injured tissues to improve the clinical benefits of MSC transplantation.

## 2. Characterization and Phenotype of MSCs

To explore the migration and homing of MSCs, first it is necessary to describe the differences among the types of MSCs and to identify the taxonomy of MSCs and the range of their environments and behaviors. MSCs are defined as multipotent cells with self-renewal capacity, capable of differentiating into a variety of cells [26]. Since the first isolation of MSCs from the bone marrow by Friedenstein and colleagues [27], MSCs have been derived from multiple tissues [2]. Since different methods have been employed to culture MSCs from multiple tissues, to assess their differentiation potential and to evaluate their capacity for self renewal, it is critical to set accepted criteria for defining MSCs. Given the lack of universally accepted criteria for defining MSCs, the Mesenchymal and Tissue Stem Cell Committee of the International Society for Cellular Therapy proposed a set of standards to define MSCs for both laboratory-based scientific investigations and preclinical studies [28]. These are: (1) plastic adherence ability; (2) lack of hematopoietic markers, such as CD45, CD34, CD14, CD11b, CD79**α**, CD19, and HLA-DR; (3) tripotential mesodermal differentiation potency into osteoblasts, chondroblasts, and adipocytes. Along with mesodermal differentiation capability, MSCs were shown to differentiate into cells of the ectodermal lineage such as neurons [29–31], keratocytes [32], and keratinocytes [33], but also into cells of the endodermal lineage such as hepatocytes [34, 35] and pancreatic *β*-cells [36]. Although the differentiation capability of MSCs into cells of the ectodermal and endodermal lineages has been demonstrated in previous studies, MSC differentiation to these lineage cells requires further investigation. Besides cellular differentiation, through an interaction with a series of signals from local tissue, engrafted MSCs can secrete diverse cytokines, possess trophic and immunomodulatory functions, and subsequently contribute to tissue repair and/or regeneration [2].

MSCs are found in various tissues and organs, including fat, periosteum, synovial membrane, synovial fluid, muscle, dermis, deciduous teeth, pericytes, trabecular bone, infrapatellar fat pad, articular cartilage, umbilical cord and cord blood [37, 38], and the placenta [39]. BMMSCs were first isolated and used in disease indications [40]. Aspirating bone marrow from patients is an invasive procedure [41] and yields only low numbers of cells (about 1–10 cells per 1 × 10^5^ cells or 0.0001–0.01% of all bone marrow nucleated cells), requiring longer and more complex *in vitro* cellular expansion procedures [42]. However, Ohgushi et al. [43] demonstrated that BMMSCs cultured from 3 mL of aspirated bone marrow obtained by noninvasive needle aspiration under local anesthesia showed therapeutic effects in treating osteoarthritis [43]. The therapeutic potential of BMMSCs was influenced by donor age, showed declining differentiation capacity, and reduced vitality *in vitro* with increasing donor age [44]. Adipose tissue is an attractive source of MSCs for stem cell therapy because it is easily obtainable in sufficient quantities by a minimally invasive procedure [45, 46]. Furthermore, adipose tissue contains more MSCs than does the bone marrow (about 100,000 MSCs per gram of fat) [47], while differentiation and immunomodulatory potencies of AdMSCs are equivalent to those of BMMSCs [46]. Of interest, a comparative study on the differentiation capability between BMMSCs and AdMSCs was performed using cells from the same donor rat. Hayashi et al. clearly demonstrated an excellent osteogenic differentiation capability of BMMSCs compared with AdMSCs derived from the same donor rat [48].

## 3. Distribution of MSCs after Systemic Infusion

The distribution and migratory properties of systemically injected MSCs is helpful in determining the metrics of homing efficiency. After intravenous delivery, MSCs are found at low or very low frequencies in most target organs, as shown by fluorescent protein labeling [49–52], transduction of MSCs with reporter genes [53, 54], detection of human genes in animal recipients [55–58], sex-linked chromosome gene for sex mismatch [59, 60], histology [51, 61], immunohistochemistry [53, 54, 56, 57], real-time PCR [49, 59], and fluorescent *in situ* hybridization [59, 60]. For instance, in baboons, by detecting transplanted cell-specific DNA, Devine et al. [49] demonstrated a high number of transplanted cells observed in gastrointestinal tissues and a relatively high number of cells also observed in the kidney, lung, liver, thymus, and skin. The levels of engraftment in these tissues were estimated, ranging from 0.1% to 2.7% of the administered cells. In noninjury models, by detecting enhanced green fluorescent protein (GFP)-transfected murine MSC, Deak et al. [52] demonstrated that the most frequently GFP-positive organs were the lungs, liver, kidney, skin, and gut among investigated tissues 24 h after MSC transplantation. However, the aforementioned methods are invasive and static, meaning the cells are not dynamically tracked. To overcome these problems, non- or minimally-invasive and efficient real-time imaging techniques are required. The development of noninvasive techniques such as magnetic resonance imaging (MRI) on superparamagnetic iron oxide (SPIO) nanoparticle-labeled MSCs [62–64], combined single-photon emission CT (SPECT)/CT scanning [65], and quantum dot tracking [66, 67] has enhanced our ability to investigate MSC homing as well as the behavior and organ-specific accumulation of transplanted MSCs. MRI cell tracking using SPIO is thought to be the lowest risk alternative for monitoring stem cell activity in humans due to the widely available data regarding the risk of MRI and the fact that SPIOs are Food and Drug Administration approved. Hsiao et al. [62] reported that MSCs were successfully labeled with Ferucarbotran, a clinically used ionic SPIO, without the aid of a transfection agent, and did not affect cell viability, proliferation, mitochondrial membrane potential change, reactive oxygen species production, or differentiation capacity. Approximately 45.2% of labeled MSCs can be detected at a single-level 3D gradient echo sequence and four repetitions using 1.5T MRI. Reagan and Kaplan [64] reviewed the details of MRI methods used to track cells and the potential and challenges for each technique in clinical translation. Using SPECT/CT imaging in an acute myocardial infarction model, Kraitchman et al. [65] demonstrated that the initial localization of BMMSCs was observed in the lung and the cells moved to nontarget organs such as the liver, kidney, and spleen within 24 to 48 h after infusion. An increase in MSCs found in the infarcted heart tissue was observed with a simultaneous decrease in the initial concentration of MSCs in the lung 24 h after infusion, and MSCs persisted until 7 days after injection. In addition, the labeling of BMMSCs with bioconjugated quantum dots does not alter the self-replication and differentiation potential of MSCs into chondrogenic, osteogenic, and adipogenic cells [66], and is very useful not only for tracking MSCs but also in investigating the behavioral changes of cells when MSCs are injected in combination with chemical compounds such as drugs like heparin [67]. By imaging mice with acute liver failure, Yukawa et al. [67] reported that within 10 min almost all transplanted AdMSCs accumulated in the lungs in the absence of heparin treatment. However, when heparin was used in combination with AdMSCs, the accumulation of the transplanted stem cells was found not only in the lungs but also in the liver, and the accumulation increased by about 30% in the injured liver. Collectively, studies using different methods for tracking MSCs have shown an initial concentration of MSCs in the lung after transfusion [52, 65, 67–69], after which most MSCs moved gradually to injured sites [52, 65, 67, 69] or to the liver, spleen, kidney, and bone marrow [68].

## 4. Migration and Homing Potential of MSCs to Sites of Injury after Systemic Infusion

The ability to regenerate damaged tissues is a common characteristic of multicellular organisms. A cycle of apoptosis and tissue regeneration exists in organisms, and stem cells in and around damaged tissues play among the most critical roles in wound healing and tissue regeneration [20]. It was generally assumed that factors released upon tissue damage or apoptosis mobilize and recruit stem and progenitor cells to the damaged site, where they proliferate and differentiate, eventually replacing the damaged tissues [22, 25]. However, a lack of data exists concerning the mechanisms driving MSC trafficking after intravenous, intraarterial, or local intra-tissue application compared with the relatively well-characterized leukocyte homing cascade [70]. Recently, Karp and Leng Teo [24] defined MSC homing as the “arrest of MSCs within the vasculature of the respective tissue,” followed by transmigration across the endothelium. Chemokines, cytokines, and growth factors released upon injury provide migratory cues for systemically or locally administered stem cells. The cues induce upregulation of selectins and activation of integrins on the stem cell surface, enabling cells to interact with the endothelium. Stem cells subsequently adhere and transmigrate across the endothelial layer into tissues. Regarding the homing capability of MSCs, numerous studies have confirmed that systemically infused MSCs can migrate to injured, inflamed tissues and exert therapeutic effects [21, 23]. BMMSCs, delivered intravenously to rats following myocardial infarction localize in the infarct region and improve ventricular function, while MSCs delivered intravenously to noninfarcted rats localize to the bone marrow [71]. In addition, localized abdomen irradiation has been shown to significantly enhance MSC homing specifically to radiation-injured tissues in mice [72]. Human AdMSCs infused by tail vein mobilized to cell-damaged areas in an allergic rhinitis animal model [73].

Evidence confirms the involvement of chemokines or growth factors as migratory cues in MSC trafficking to the injured region [24]. The interactions of stromal cell-derived factor-1*α* (SDF-1*α*) and C-X-C chemokine receptor type 4 (CXCR4) were found to mediate the trafficking of transplanted BMMSCs in a rat model of left hypoglossal nerve injury. Inflammatory cytokines, transforming growth factor (TGF)-*β*1, interleukin (IL)-1*β*, and tumor necrosis factor (TNF)-*α* upregulate the production of matrix metalloproteinases (MMPs) in MSCs, resulting in a strong stimulation of chemotactic migration through the extracellular matrix, while the chemokine SDF-1*α* exhibited minor effects on MMP/tissue inhibitor of metalloproteinase (TIMP) expression and cell invasion [74]. BMMSCs are mobilized by chemokines that are present in the supernatants of primary cultures of human pancreatic islets culture *in vitro* and *in vivo* [75]. Human AdMSCs migrate in response to a variety of growth factors and cytokines including platelet-derived growth factor (PDGF)-AB, TGF-*β*1, TNF-*α*, and SDF-1*α* [76]. Of interest, in a previous study, human AdMSCs pre-stimulated with TNF-*α* showed enhanced migratory activity compared to the nonpretreated control group [76]. These results indicate that enhancement of the homing capacity of MSCs can be achieved by modulating the response of MSCs to a variety of growth factors and cytokines, thereby improving their therapeutic potential.

## 5. Homing Strategies to Enhance Efficacy and Safety of MSC Therapy

Locally or systematically introduced MSCs have been used for cellular therapy for a variety of indications. BMMSCs have been used in a number of published interventions for a range of therapeutic applications [77, 78]. Among other applications, BMMSCs have been used to reduce clinical symptoms of osteogenesis imperfecta [79] and to treat large bone defects [80], in regenerative treatments to enhance repair of pancreatic islets [81], and in infarcted myocardium [82–84]. Furthermore, BMMSCs have been applied in a variety of immunomodulatory treatments of autoimmune diseases, including Crohn's disease [85, 86], multiple sclerosis (MS) [87], and rheumatoid arthritis (RA) [88].

Like BMMSCs, AdMSCs have been demonstrated in clinical trials to be safe and suitable for introduction into the human body following culturing [89–91]. Local or systemic administration of AdMSCs was reported to have therapeutic efficacy in treating myocardial infarction [92], liver injury [93], hypoxia-ischemia-induced brain damage [94], allergic rhinitis [73], and muscular dystrophy [95]. Furthermore, the immune regulatory ability of AdMSCs has warranted their therapeutic application to treat immune-related diseases including graft versus host defense (GVHD) [96], rheumatic disease [97], and thyroiditis [98]. Systemic infusion of AdMSCs before transplantation of haploidentical hematopoietic stem cells (HSCs) controls lethal GVHD reaction of allogenic HSCs in mice [96]. Human AdMSCs reduced disease severity in experimental autoimmune thyroiditis via downregulation of Th1 cytokines and improved Th1/Th2 balance [98]. In humans, systemic administration of autologous human AdMSCs is a promising alternative to treat patients with autoimmune diseases including autoimmune ear disease, MS, polymyositis, atopic dermatitis, and RA [99]. In each of these therapeutic applications, the ability of stem cells to home to the site of injury was critical to their *in vivo* effects on the symptoms or underlying pathologies of these diseases.

Homing may provide an important clinical application of MSCs in the future as a cellular vehicle for anticancer therapeutics in tumors [89]. Maestroni et al. [100] reported that BMMSCs induced significant reductions in size and metastasis of lung cancer cells or melanoma cells in mice. Because tumors release a range of cytokines and preferentially recruit MSCs, stem cells may be used to deliver antitumor drugs in a preclinical setting. Studeny et al. [101, 102] demonstrated that BMMSCs transfected with IL-1*β* migrated to tumors and exerted an anticancer effect by secreting IL-1*β*. Khakoo and his team [103] reported that a single injection of human BMMSCs into the tail vein of immunocompromised mice bearing Kaposi's sarcoma suppressed tumor growth by more than 50%, and two injections suppressed the growth even further. Hakkarainen et al. [104] loaded MSCs derived from bone marrow and adipose tissue with oncolytic adenovirus and injected the stem cells into the tail vein of mice bearing lung and breast cancer cells. The authors found that the stem cells did not home to tumors but increased the therapeutic efficacy in lung and breast cancer cells compared to the control group injected with the virus alone. In 2007, Kucerova et al. [105] subdermally and systemically injected AdMSCs overexpressing a cancer cell cytotoxic prodrug, cytosine deaminase (CD), into mice bearing HT-29 colon cancer. Direct migration of CD-AdMSCs to the colon cancer cells was observed *in vitro*, and a significant inhibition of tumor growth was observed by subcutaneously or intravenously administered CD-AdMSCs in immunocompromised mice treated with 5-fluorocytosine. Qiao et al. [106] reported that human MSCs significantly inhibited the proliferation, colony-forming ability, and oncogene expression growth in malignant liver cancer cell lines, H7402 and Hep2, both *in vitro* and *in vivo* through Wnt signaling pathway. There were no cases of recurrence during the 100-day observation period that followed. In 2009, Cousin et al. [107] demonstrated that human AdMSCs strongly inhibit the proliferation of pancreatic ductal adenocarcinoma both *in vitro* and *in vivo* through altering cell cycle progression, thereby inducing tumor cell death. Canine adipose-derived stem cells loaded with interferon-*β* in combination with an anticancer drug, cisplatin, was shown to inhibit the growth of melanoma cells in mice [108].

Although there may be a plateau between the number of delivered cells and improvement of clinical outcome [54], a higher number of infused MSCs are expected to give rise to a higher number of engrafted MSCs and better functional outcomes [109, 110]. Below, several factors that affect the homing potential of MSCs will be discussed, including the quality of MSCs *per se*, the ability of MSCs to respond to migratory stimuli, the physiological barrier blocking MSC migration, and the inflammatory microenvironment of the body. A variety of strategies are suggested to enhance the homing of MSCs given their known homing characteristics.

### 5.1. Cultivating MSCs with Enhanced Migratory Ability by Optimizing Cell Culture Conditions

Highly active MSCs or progenitors are naturally attracted to signals that come from sites of injury [25]. Thus, the culture process of MSCs should maintain the characteristics of the donor/recipient's MSCs, that is, their homology. It has been demonstrated that cell culture conditions including the passage number, confluency of the passaged cells, and oxygen concentration have a significant impact on the expression of cell surface receptors of MSCs responding to migratory signals. The passage number of MSCs affects homing as MSCs have been shown to gain or lose certain surface receptors during culture. Freshly isolated MSCs display enhanced homing ability compared to their culture-expanded counterparts [111]. Homing receptors CXCR4, a chemotactic receptor for SDF-1*α* that is upregulated in the bone marrow and in ischemic tissues, is usually absent on the surface of culture-expanded MSCs [8, 112–114]. However, treatment of MSCs with a cocktail of cytokines in culture has been shown to induce high surface expression of CXCR4 [115].

The confluency of cultured MSCs prior to therapeutic infusion also affects migration potential. Lee et al. [116] investigated the differences between low-passage and low-density cultures versus MSCs from expanding, near-confluent cultures. Six surface markers were found preferentially expressed on early passage MSCs in low confluency cultures: podocalyxin-like protein PODXL, CD49f, CD49d, cMet, CXCR4, and CX3CR1. Sorting PODXLhi/CD49fhi cells with specific antibodies resulted in selection of early MSC progenitors that were less prone to produce lethal pulmonary emboli and increased homing to the heart in a murine myocardial infarction model. De Becker et al. [117] demonstrated that high culture confluence inhibited transendothelial migration in MSCs by increasing the production of a natural matrix MMP inhibitor, TIMP-3.

The oxygen level in cell culture conditions also influences cell homing [118, 119]. Exposure of MSCs to hypoxic conditions increased CXCR4 and CX3 chemokine receptor 1 (CX3CR1) expression, which leads to increased migration in response to SDF-1*α*. Grafting experiments using xenotypic chick embryo showed that cultured MSCs under hypoxic conditions engrafted more efficiently compared with cells from normoxic cultures [119]. Rosová et al. [120] demonstrated that MSCs cultured in hypoxia activated the Akt signaling pathway while maintaining their viability and cell cycle rates. Hypoxic preconditioning also induced expression of cMet, the major receptor for hepatocyte growth factor, and enhanced cMet signaling. Migration rates are also increased in hypoxia, and hypoxic preconditioning increased MSC migration in Matrigel by upregulating MMPs [121].

Although a number of strategies have been discussed to improve culture conditions, the most critical aspect for clinical application of MSCs is the safety of cultured cells. *Ex vivo* expansion of MSCs for long-term culture alters the characteristics of MSCs, including their proliferative capacity [122], differentiation potential [123], and trophic activity [124]. We previously provided substantial guidelines for evaluating the safety of cultured MSCs by conducting *in vitro* and *in vivo* assays under good laboratory practices [91]. These assays include sterility, immunophenotyping, differentiation potential, genetic stability test, *in vivo* toxicology, and tumorigenicity tests in laboratory animals and *in vivo* safety tests in the spinal cord of patients receiving 400 × 10^6^ stem cells intravenously.

### 5.2. Enhancing the Ability of MSCs to Respond to Migratory Stimuli

To respond to migratory signals released in sites of injury, MSCs must express surface receptors capable of sensing those signals. Various studies to modify MSCs or to enhance expression of surface markers have been explored to enhance MSC migration. A key player in MSC migration is the CXCR4-SDF-1*α* axis [24]. Many studies have focused on ways to enhance the functional expression of CXCR4 in MSCs to migrate toward chemotactic SDF-1*α* secreted at injury sites. Modification of CXCR4 expression with retroviral overexpression, mRNA transfection of CXCR4-GFP [125], and cytokine pretreatment especially TNF-*α* resulted in increased migration toward SDF-1*α* *in vitro* [76, 126]. Maijenburg et al. [127] investigated gene expression profiles involved in the process of MSC migration and found 12 differentially expressed genes in migratory MSCs compared to nonmigrating MSCs. Among them, the nuclear receptors Nur77 and Nurr1 showed the highest expression in migratory MSCs. The expression of these receptors rapidly increased under stimulation with SDF-1*α* and PDGF-BB. Genetically engineered MSCs overexpressing Nur77 or Nurr1 showed enhanced migration toward SDF-1*α* and decreased cell proportion in S-phase cell cycle. Monocyte chemoattractant (MCP)-1 is typically expressed at sites of inflammation and can thus represent a model homing chemokine [128]. GFP-labeled MSCs expressing the MCP-1 receptor, chemokine receptor (CCR) 2, on the cell surface were systemically infused into transgenic mice expressing MCP-1 specifically in the myocardium. A higher frequency of GFP-positive cells (20 cells/microscopic field) was observed in the myocardium of the transgenic mice compared to the hearts of control mice 7 and 14 days later. In another study, the upregulation of the *α*4 subunit of the VLA-4-integrin on MSCs using an adenovirus vector resulted in successful dimerization with *β*1-integrin and increased the homing ability of MSCs to the bone marrow by more than 10 fold as compared to nontransduced MSCs [129]. Since human MSCs do not express E-selectin ligands, Sackstein and colleagues enzymatically modified the native CD44 glycoform on MSCs into hematopoietic cell E-selectin/L-selectin ligand, resulting in increased MSC migration to the bone marrow [117].

### 5.3. Modulating Physiological Barriers Blocking MSC Migration into the Site of Injury

Noninvasive administration of stem cells is more convenient and compassionate than invasive methods for cell therapy, particularly when the patient suffers from degenerative conditions or has an autoimmune indication. Blood vessels are the primary route through which MSCs circulate in the body. The vessels need to be clear of debris and broken capillaries must be fixed in order for the cells to travel to the appropriate sites in the body. Thus, the body's revascularization capacity must be adequate. If the injured area is in need of further therapy, more infused cells may be required. In these cases, the blood vessels must be clot-free, allowing cells to reach the area of injury during subsequent infusions. Furthermore, MSCs must pass through physical lung barriers and transmigrate into the tissue of injury. To solve the problems induced by microvessels and the lung, Yukawa et al. [67] proposed a combination of MSCs and heparin. When only MSCs were injected systemically in the mice, almost all transplanted MSCs were accumulated in the lungs. However, when the mice were treated with heparin, accumulations decreased in the lung and increased in the acute injured livers of these mice.

When cultured MSCs are infused into the body, what kinds of conditions can impair the ability of cells to reach their final location at the injury site? Modulating the harmful environment can improve the migration of MSCs into target tissues. Sites of tissue damage undergo chronic or acute immune responses, and MSCs migrating to these sites will encounter various immune cells in the local environment. Thus, MSC cellular regeneration can be influenced by immune cells in the damaged sites [130]. Liu et al. [130] presented findings that provide novel insight into how the host immune systems communicate via interferon (IFN)-*γ* and TNF-*α* with transplanted MSCs during bone formation and repair. The authors demonstrated that systemic infusion of regulatory T cells or local administration of aspirin markedly enhanced the survival of BMMSCs and improved bone regeneration of transplanted MSCs through suppression of IFN-*γ* and TNF-*α* in damaged bone sites.

### 5.4. Stimulating the Target Site to Recruit MSC Mobilization

In the acute phase of injury, factors released from damaged tissues recruit blood cells and MSCs to repair the injured site. In this regard, substantial evidence indicates that infused MSCs have higher engraftment efficiencies within sites of inflammation or injury. François et al. [58] applied total body irradiation (TBI) or TBI in combination with additional local irradiation into the abdominal area or hind leg of mice. The authors found that the engraftment level of systemically infused MSCs was higher in mice subjected to TBI compared to nonirradiated mice. Cho et al. [73] also demonstrated that inflammatory stimuli of allergic rhinitis induced the homing of intravenously administered hAdMSCs to cell-damaged areas. Taken together, the evidences indicate that signals are required to recruit MSCs with high efficiency, which is critical for improving the clinical benefits of MSCs. However, in a variety of clinical indications, MSCs are administered to damaged tissues at the subchronic or chronic phases of injury, in which the migratory signals for MSCs may be minimal or absent. Thus, exogenous stimuli are necessary to recruit infused MSCs into subchronic or chronic phases of injury sites for high efficacy of MSC therapy.

In this regard, electric stimuli can be a good candidate directing MSCs migration to injured sites. Evidence demonstrates that electrical stimulation (ES) induces the migration and stimulation of adult cells, including stem cells, and improves the clinical benefit. Electrical current applied to wounded tissue activates and migrates fibroblasts, which play a critical role in wound healing [131, 132]. Gardner et al. [133] performed a meta-analysis on the effect of ES on chronic wound healing in human patients and reported that ES increased the rate of chronic wound healing to 144% that of the control in 24 studies. In an animal model of spinal cord injury, application of an electrical field (EF) resulted in functional improvement [134]. On the basis of the effects of EFs on functional improvement in animal models of spinal cord injury (SCI), Shapiro et al. [135] applied weak EF stimulation in human SCI in a phase I trial and reported considerable clinical benefit. Perry et al. [136] applied degenerate electrical waveforms in the treatment of skin scarring in 30 patients with over 140 scars with long-term pain and itching. After monitoring for 6 months, the authors reported that ES treatment resulted in a clinically and statistically significant reduction of symptoms and scar scores. Zhao et al. [137] demonstrated that physiological EF of ~25 mV/mm *in vitro* directed the migration of cultured BMMSCs mainly to the anode. Increasing the EFs enhanced the migration of the MSC and peaked the response at 300 mV/mL at a rate of 42 ± 1 *μ*m/h, around double the migration rate of the control (no EF). Of importance, EF did not influence cell senescence, phenotype, or the osteogenic potential of MSCs, regardless of passage number within the range tested (P3–P10). Recently, it has been demonstrated the combination of MSC transplantation with ES can be a therapeutic tool to improve the efficacy of transplantation. Wu et al. [138] demonstrated that implanted spike wave ES improved the survival of BMMSCs after transplantation compared to BMMSCs transplantation or ES treatment alone using an *in vivo* rat model of spinal cord injury. Furthermore, analysis of functional parameters demonstrated improved functional recovery in the BMMSCs + ES groups. Taken together, it can be hypothesized that applying ES around injured sites can direct migration of exogenously infused MSCs and enhance the migration of MSCs during the healing process. This hypothesis requires further verification.

## 6. Conclusions

The efforts of researchers to establish the safety of MSC infusion and their effects *in vivo* have led to the application of MSCs for the treatment of various tissue degenerative indications in humans [99]. Thus far, most of the procedures involve local administration or direct injection [http://www.clinicaltrials.gov/]. However, for conditions such as Alzheimer's, Parkinson's, liver disease, renal failure, and autoimmune diseases, the delivery of MSCs by systemic infusion can be minimally invasive and convenient. To make a systemic infusion efficacious, more MSCs are needed by comparison to local delivery. MSCs can be expanded via *in vitro* culture, which unfortunately presents high costs. Thus, further research is required to understand the factors affecting the efficiency of MSC migration and to determine strategies to remove harmful factors and improve homing of MSCs to the area of injury. New strategies could mean smaller quantities of MSCs necessary for infusion, thereby attaining the intended therapeutic goal with greatest efficiency and efficacy. To achieve this goal, cell migration and tracking studies must be conducted in various *in vivo* environments along with *in vitro* laboratory studies. Through these studies, optimized culture conditions can be established to cultivate MSCs with enhanced homing ability and expressing the appropriate homing receptor. This is also essential to improve vascular conditions, so that introduced cells can easily migrate to damaged sites. In addition, it is critical to determine exogenous stimuli such as ES to recruit infused MSCs into the subchronic or chronic injury sites. Ultimately, the future of stem cell therapy depends, as does so much of science in general, on understanding the nature of responses to illness. Just as bees are naturally attracted to flowers and men are attracted to women, the philosophy of research of MSC migration and homing should focus on the nature of life.
